# Editorial: Functionalized Nanocarriers for Theranostics

**DOI:** 10.3389/fbioe.2020.616574

**Published:** 2020-11-10

**Authors:** Stefano Leporatti, Andrea Ragusa, Rawil Fakhrullin

**Affiliations:** ^1^Consiglio Nazionale delle Ricerche CNR-Nanotec, Istituto di Nanotecnologia, c\o Campus Ecotekne, Lecce, Italy; ^2^Department of Biological and Environmental Sciences and Technologies, University of Salento, Lecce, Italy; ^3^Institute of Fundamental Medicine and Biology, Kazan Federal University, Kazan, Russia

**Keywords:** nanocarriers, theranostics, nanomedicine, nanosystems, nanoplatforms

Nanotechnology has led to the development of a variety of nanocarriers with applications in diagnosis and therapy, giving rise to novel theranostic nano-tools. They combine diagnostic and therapeutic moieties into a single nano-device, potentially allowing an early detection of the pathology together with targeted treatment.

Our Research Topic has gathered several contributions describing novel functionalized nano-transporters, such as targeted nanoparticles (NPs), nanovesicles (NVs), liposomes (LPs), and nano-clays, for combined diagnosis and therapy. Eleven articles have been collected with a well-balanced ratio: five are comprehensive reviews and the other six are original research articles.

Among a plethora of different nanomaterials available to fabricate drug delivery systems (DDSs) for cancer therapy and diagnosis, poly(D,L-lactic-*co*-glycolic acid) (PLGA) has been extensively used due to its biocompatibility and biodegradability. In a comprehensive review article, Shen et al. report the employment of PLGA-based DDSs for remotely triggered cancer therapy, including photo-triggered, ultrasound-triggered, magnetic field-triggered, and radiofrequency-triggered cancer therapy, involving photodynamic therapy (PDT), photothermal therapy (PTT), and photo-triggered chemotherapeutics release. The state-of-the-art in theranostic approaches involving mesoporous silica nanoparticles (MSNs) for treating hepatocellular carcinoma (HCC) has been reviewed by Tao et al. In this work, they outline the recent advances in MSNs-based systems for HCC therapy and diagnosis and they also discuss the precision delivery strategies of MSNs in liver cancer.

The signature service and insoluble network formation of the peptide self-assemblies as hydrogels have drawn a plethora of research activity among scientists all over the globe in the past decades. In this respect, Gupta et al. review the last 5-year efforts on novel approaches for the design and development of single molecule amino acids, ultra-short peptide self-assemblies (di- and tri-peptides only) and their derivatives as drug/gene carriers and tissue-engineering systems. Peptides and small molecule-based nanostructures can be convenient alternatives for therapeutic delivery due to their good biocompatibility and their easy design, synthesis, and functionalization. These properties render their self-assembled nanostructures smart tools suitable for biomedical applications (Gupta et al.).

Parodi et al. overview the recent development of smart nanotheranostic systems responsive to pathological stimuli, including oxidative stress, altered pH, enzymatic expression, and reactive biological molecules. Therapeutic and diagnostic properties can be included in the same molecule embedded in the nanocarrier structure that can be activated at the injury site, or they can independently derive from different chemicals loaded into and/or conjugated onto the surface of the NPs.

Currently, the onset of neurodegenerative diseases (NDs) is strongly widespread due to the increasing age of the world population and the lack of efficient therapies. In a state-of-the-art review article, Cascione et al. overview the lipid-based drug delivery improvements in *in vivo* applications against NDs. Lipid carriers have a good ability to deliver both hydrophobic and hydrophilic molecules through the Blood-Brain Barrier (BBB), demonstrating an enhanced efficacy of the drug following liposomal encapsulation.

The six original research articles contributions describe novel tailored nano-carriers for drug delivery applied to cancer and neurodegenerative diseases treatment. Two of them are dealing with the use of functionalized halloysite nanotubes (HNTs) as efficient drugs/active molecules nano-transporters (Guryanov et al.; Saleh et al.). Guryanov et al. report on prodigiosin-loaded halloysite-based nanoformulation (p-HNTs) and its effects on cell viability. By comparing the effects of p-HNTs on malignant (Caco-2, HCT116) and non-malignant (MSC, HSF) cells, the authors demonstrate selective cytotoxic and genotoxic activity. Moreover, in another very interesting and prospective article, Saleh et al. proposed the use of HNT nanocarriers to penetrate the BBB and effectively deliver the payload over an extended time period. On the other hand, the Sukhorukov's group tried to address the delivery of neuropeptides on-demand, potentially suitable for the migratory or axonal guidance of human nerve cells, by using polylactic acid (PLA)-based microchamber arrays (MCAs) (Sindeeva et al.). Optical targeting of microchambers for drug release triggered functional cell response locally.

The last three contributed articles are reporting the use of different type of NPs for delivering and releasing drug against different type of tumors. In this respect, Zacheo et al. developed lipid NVs of size varying from 100 up to 300 nm and successfully loaded them with fluorophores molecules (DOP-F-DS and a fluorescent protein), inorganic nanoparticles (quantum dots and magnetic NPs), and anti-cancer drugs (SN-38 and doxorubicin).

The synthesis and the functionalization of gold NPs with cancer-specific biomolecules may represent a winner strategy for a selective and targeted tumor-phototherapy. With this in mind, Bloise et al. described a simple approach for the synthesis of extra-small gold NPs for breast cancer therapy. Extra-small gold nanospheres stabilized with a thiol-functionalized polyamidoamine (AGMA1) and trastuzumab were synthesized and tested *in vitro* as nanovectors for breast cancer targeted drug delivery. They proposed to assemble small Au NPs into larger structures through controllable interparticle interactions by using AGMA1, with the final aim to enhance the absorption of NIR light (Bloise et al.).

To improve chemo-drug therapeutic efficiency and overcome issues such as inadequate response rates, high toxicity, severe side effects due to non-specific targeting of anti-cancer drugs, and the development of multidrug resistance during prolonged treatment, a multifunctional NP was developed to effectively target and treat melanoma by Yaman et al.. PLGA NPs were coated with a cellular membrane derived from the T-cell hybridoma, 19LF6 endowed with a melanoma-specific anti-gp100/HLA-A2 T-cell receptor (TCR) and loaded with trametinib, an FDA-approved melanoma chemotherapeutic drug.

## Author Contributions

SL wrote the editorial, which was revised, proofed, and accepted by all the authors.

## Conflict of Interest

The authors declare that the research was conducted in the absence of any commercial or financial relationships that could be construed as a potential conflict of interest.

